# A rare presentation of asymmetric limb hypertrophy and diffuse capillary malformations in a pediatric patient with Loeys-Dietz syndrome type 3

**DOI:** 10.1016/j.jdcr.2023.10.008

**Published:** 2023-10-31

**Authors:** Nouf Almuhanna, Sarah Alkhezzi, Rasha Alhamazani, Mohammed Aljughayman, Bushra Saeed Alasmari, Abdulaziz Sultan Alsuhibani, Faris A. Alhomida

**Affiliations:** a Department of Dermatology, King Fahad Medical City, Riyadh, Saudi Arabia; b College of Medicine, King Saud bin Abdulaziz University for Health Sciences, Riyadh, Saudi Arabia

**Keywords:** congenital anomalies, genodermatosis, Klippel-Trenaunay syndrome, Loeys-Dietz syndrome, pediatric, pediatric dermatology, port-wine stain, Sturge-Weber syndrome, vascular malformation

## Introduction

Loeys-Dietz syndrome (LDS) is a rare, aggressive, heterogeneous group of autosomal dominant, genetic disorders characterized by multisystem connective tissue abnormalities and vascular manifestations, primarily affecting the aortic and arterial vasculature. LDS has also been associated with craniofacial and ocular abnormalities, and cutaneous manifestations, including thin, velvety skin, easy bruising, visible or varicose veins, and dystrophic scarring. LDS is subclassified into 5 subtypes based on the pathogenic variant (PV) identified, with LDS type 3 (LDS3) reported to be caused by a heterozygous PV in the SMAD3 gene on chromosome 15q and is associated with early-onset osteoarthritis.^1^, ^2^, ^3^, ^4^

## Case presentation

A 7-month-old normocephalic girl with a history of cataracts, seizures, and known to have both Sturge-Weber syndrome and LDS3 was referred to our dermatology clinic for further management of diffuse capillary malformations (CMs) since birth. She was born late preterm at 36 weeks and 6 days, and was delivered via emergency C-section due to decreased fetal movements and breech presentation to consanguineous parents. Her history is notable for a prolonged 21-day stay at the neonatal intensive care unit for respiratory distress. Family history was notable for maternal hypothyroidism. She has no known drug allergies and is currently taking oral 20 mg aspirin once-daily, oral 90 mg oxcarbazepine twice-daily, and oral 12 mg topiramate twice-daily. Genetic testing done at an outside facility before presentation revealed a heterozygous PV of the SMAD3 gene on chromosome 15q, thus rendering the diagnosis of LDS3. She is the sole family member affected. The parents, showing no LDS features, declined parental segregation testing.

**Fig 1:**
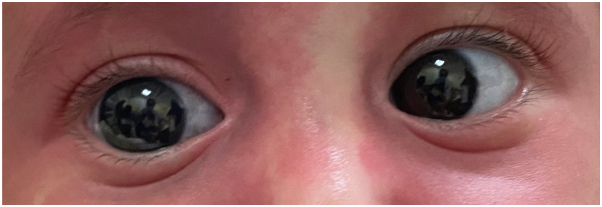
Blue sclera of the bilateral eyes are noted.

**Fig 2:**
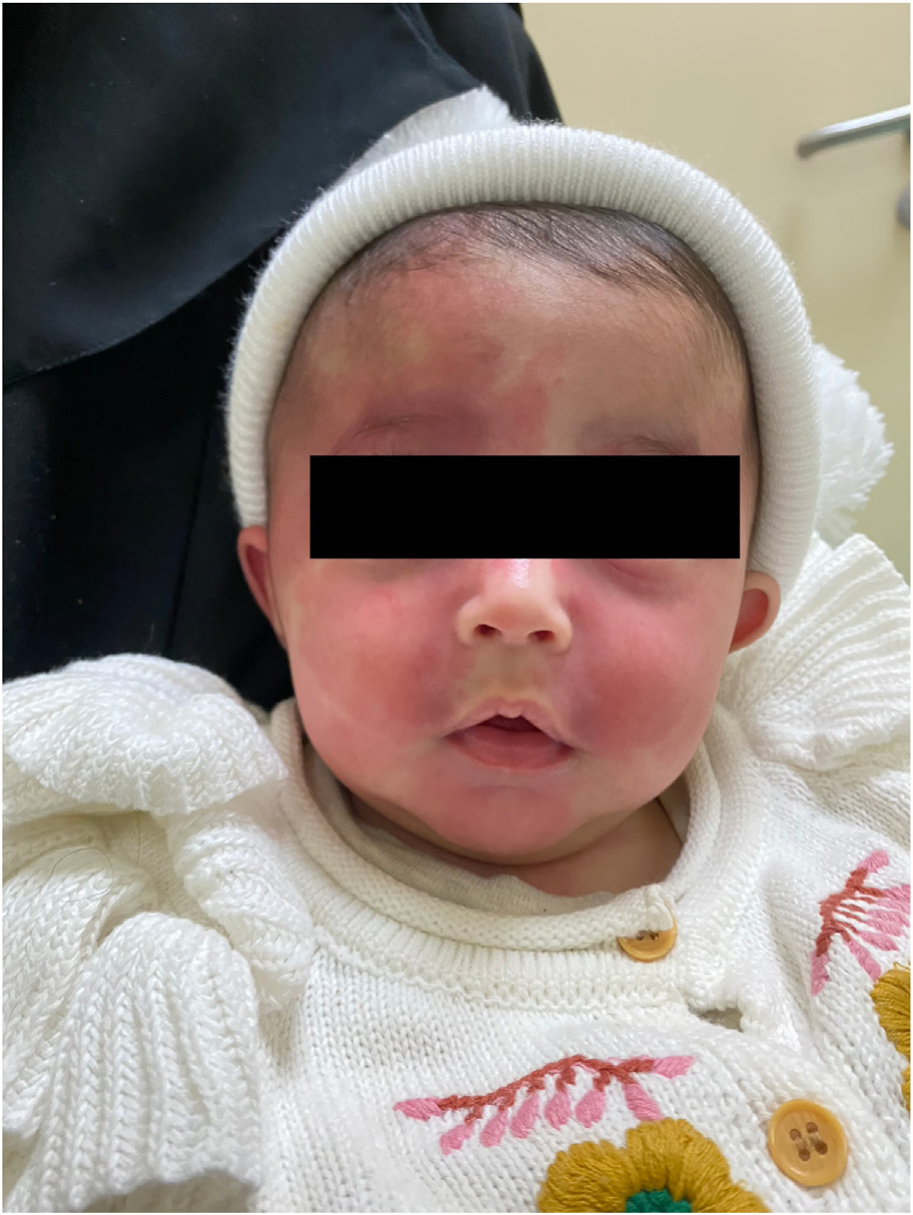
Confluent, erythematous patches of the forehead, bilateral cheeks, and chin are noted.

**Fig 3:**
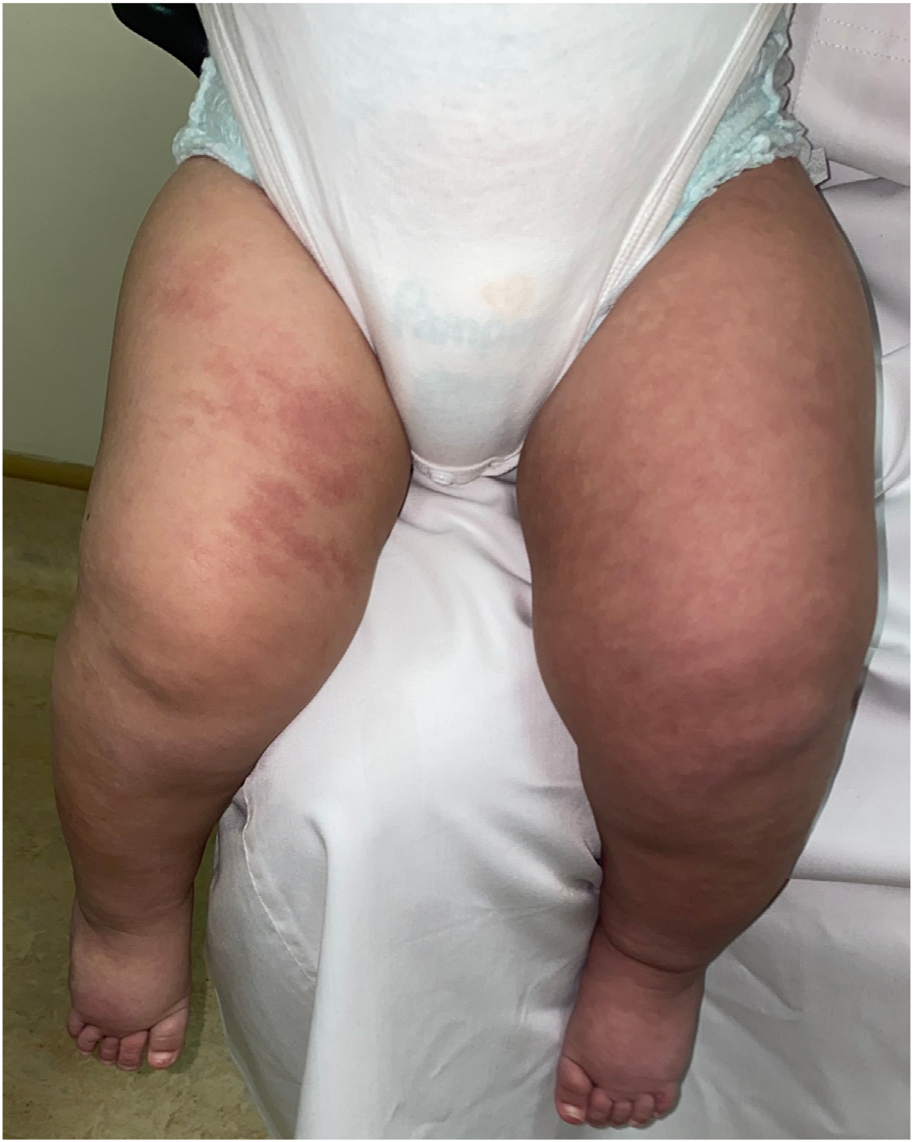
A photograph of the patient’s asymmetric and hypertrophied left side of the lower limb with overlying, scattered, erythematous, confluent, and reticulated patches of the bilateral lower extremities.

Examination revealed a unilateral, confluent, erythematous patch along the V1 distribution of the right side of the forehead, bilateral, confluent, erythematous patches along the V2 and V3 distribution of the cheeks and chin, bilateral blue sclerae, and diffuse, reticulated, confluent, erythematous patches of the chest, abdomen, back, groin, and bilateral lower extremities (Fig 1, Fig 2, Fig 3). The left side of the lower extremity was notably more hypertrophied than the right (Table I and Fig 3). However, there was no limb-length discrepancy noted of the upper or lower limbs. Furthermore, there were no lipomas, epidermal nevi, café-au-lait macules, dermal melanocytosis, nevus spilus, nevus anemicus, lymphatic malformations, varicose veins, or cutis marmorata noted on examination.

**Table I:** Measurements of the patient at presentation and at 6-month follow-up

Body areas measured	Presentation		6-month follow-up	
Head circumference	40 cm		44.5 cm	
	Right	Left	Right	Left
Upper portion of the thigh	22 cm	27 cm	28.5 cm	34.5 cm
Patella	19 cm	22 cm	24 cm	26 cm
Lower portion of the leg	17 cm	19 cm	22 cm	25 cm

Given the lack of other findings, the diagnoses of CLOVES syndrome and phakomatosis pigmentovascularis were ruled out. By 6-month follow-up, both lower limbs grew similarly, as expected in an infant, but the left was noticeably larger than the right on examination.

Magnetic resonance imaging of the brain revealed a mild, reduced right cerebral hemisphere volume with prominent right-side cortical veins and accelerated myelination and asymmetrical dilation of the right lateral ventricle. Furthermore, MRI of the left side of the lower extremity, revealed a lipomatous muscular hypertrophy with subcutaneous edema suggestive of Klippel-Trenaunay syndrome. The presence of diffuse, superficial, serpiginous, signal-void structures suggestive of varicose veins was noted. Of note, there were no skeletal abnormalities noted. MRI of the right side of the lower extremity was unremarkable. An echocardiogram, skeletal survey, neonatal head ultrasound, neonatal spinal, and lumbosacral ultrasounds were unremarkable. Although, these findings may suggest Klippel-Trenaunay syndrome as the possible diagnosis, diffuse capillary malformation with overgrowth cannot be dismissed. The parents chose conservative management and refused a biopsy to identify a possible somatic mutation that could confirm the diagnosis.

Education, reassurance, and discussion of possible treatment options were given to the parents. The parents elected to start pulsed-dye laser treatment for the management of her CM. She continues to follow-up with pediatric dermatology, ophthalmology, neurology, cardiology, and orthopedics.

## Discussion

LDS is an exceedingly rare entity believed to be caused by PVs in the genes encoding for the proteins involved in the transforming growth factor-beta signaling pathway, leading to dysregulated transforming growth factor-beta signaling. There are currently 5 recognized subtypes of LDS, each associated with distinct genetic PVs and represent a phenotypic spectrum of severity with LDS type 1 being the most severe and LDS type 5 being the least. LDS type 1 and LDS type 2 are reported to be the most severe subtypes, and are associated with PVs in the TGFBR1 and TGFBR2 genes, respectively. LDS3, LDS type 4, and LDS type 5 are considered less severe subtypes, linked to PVs in the SMAD3, TGFB2, and TGFB3.^1^, ^2^, ^3^, ^4^, ^5^ Furthermore, a novel PV has been identified in the SMAD2 gene, consistent with phenotypic features of LDS and has been proposed as LDS type 6 but is not yet widely recognized.^2^^,^^3^^,^^6^

LDS has been reported to behave more aggressively than other similar syndromes, namely, Ehlers-Danlos syndrome and Marfan syndrome due to an earlier-onset of complications arising from aortic or arterial dissections.^1^, ^2^, ^3^, ^4^, ^5^ Although LDS portrays a wide phenotypic variety and may present with tortuous arteries and varicose veins, to our knowledge, there are no reports of LDS associated with CM, Sturge-Weber syndrome, diffuse capillary malformation with overgrowth, or Klippel-Trenaunay syndrome.^2^, ^3^, ^4^ Transforming growth factor-beta signaling is essential for the development and integrity of blood vessels; alteration of this pathway have been implicated in the development of various vascular diseases including, aortic aneurysms and arteriovenous malformations. We propose that impairment of this pathway may help explain our patient’s finding of diffuse CM in association with LDS3.^7^ However, it is unclear if this is a true association or an independent finding. Although, our findings may suggest a novel association, more research is warranted to confirm this possible association and to further elucidate the genotype-phenotype correlations and develop targeted therapeutic strategies for these complex disorders.

LDS presents as a treatment challenge as there are no treatments currently available that are approved by the Food and Drug Administration. The increased predisposition to aortic aneurysms and dissections and the heterogeneity of the disorder, necessitates a multidisciplinary treatment approach for early detection and prevention of complications. LDS patient management involves regular cardiovascular imaging, preventive surgery and pharmacological therapies with beta-blockers or angiotensin receptor blockers to mitigate aortic growth. Additionally, specialist treatments for skeletal, ocular, and cutaneous issues are required, thus emphasizing the need for a coordinated care team for optimal patient outcomes.^1^^,^^2^^,^^4^^,^^5^

In conclusion, consider genetic testing, a multidisciplinary approach, patient education and regular follow-up in a patient that presents with a myriad of findings in association with asymmetric limb hypertrophy and diffuse CM.

## Conflicts of interest

None disclosed.
